# T29. ELECTRORETINOGRAPHIC RESPONSE IN YOUTHS AT GENETIC RISK OF SCHIZOPHRENIA AND BIPOLAR DISORDER AND IN NORMAL CONTROLS: TRANSVERSAL AND LONGITUDINAL DIFFERENCES AND IMPLICATIONS FOR THE RISK TRAJECTORY

**DOI:** 10.1093/schbul/sby016.305

**Published:** 2018-04-01

**Authors:** Anne-Marie Gagné, Thomas Paccalet, Valérie Jomphe, Daphné Lussier, Michel Maziade

**Affiliations:** Centre de Recherche CIUSSS-CN

## Abstract

**Background:**

Visual defects have been widely reported in major psychosis. This includes altered eye tracking, retinal thinning and electrophysiological anomalies.1 One of the most replicated alterations is decreased electroretinographic (ERG) responses that are observed in both bipolar disorder and schizophrenia. Our previous study showed a diminished rod b-wave amplitude in a small sample of children born to an affected parent.2 The fact that an anomaly found in patients would also be observed in children at genetic risk suggests a neurodevelopmental origin and may represent a vulnerability marker. Little data exists on the stability of ERG measures in childhood and adolescence. We wanted to evaluate rod and cone ERG response in larger samples of young offspring of an affected parent (HR) and age and gender balanced controls. By comparing a subsample of 33 offspring to controls, we were able to evaluate the stability and change of ERG over time.

**Methods:**

ERGs of 71 offspring (mean age of 19 y.o.) and 224 healthy controls (mean age of 20 y.o.) was recorded. From this sample, 33 HR and 26 healthy controls had ERG recordings at 2 different moments (mean interval of 4 years). We then compared the amplitudes obtained at Time 1 and Time 2 in order to assess whether the ERG amplitudes remained stable or varied over time.

**Results:**

Congruent with our 2010 report, this larger HR sample showed a reduced rod b-wave amplitude (p<.05). Probably due to higher statistical power, two other differences were found: an increased cone b-wave latency (p<.05) as well as a diminished mixed rod/cone ERG amplitude (p <.05). None of the ERG amplitudes of the healthy controls changed over time. In contrast, 12 out of 33 HR participant showed a variation of more than one standard deviation (either increase or decrease) on the rod b-wave amplitude which was significantly more frequent than in healthy controls (2/26; p<.05). Change in offspring occurred in both directions: some of them had an increased ERG amplitude response that was sufficient to end up in the confidence interval of the controls whereas others experienced a decreased of their rod amplitude over time.

**Discussion:**

These young high-risk offspring displayed three ERG anomalies that we have already reported in adult patients.2 Our finding bolstered the evidence that ERG anomalies observed in patients may have neurodevelopmental or childhood roots. We observed only little variation in the ERG of the healthy controls over time in that early age range and this appears concordant with existing literature. Of particular interest is the finding that rod b-wave amplitudes were diminished in patients and in offspring. The offspring also showed increased variability over time in comparison to controls. Future studies will seek to understand the relationship between the transversal or longitudinal patterns of rod b-wave amplitudes, as an indicator of risk, and the risk endophenotypes previously reported in the children born to an affected parent.3,4

**References:**

1. Gagné et al. Prog Neuropsychopharmacol Biol Psychiatry. 2015

2. Hébert et al. Bill Psychiatry, 2010

3. Maziade, N Eng J Med, 2017

4. Paccalet et al., Schizophr Res, 2016

